# Endoplasmic Reticulum-Shaping Atlastin Proteins Facilitate KSHV Replication

**DOI:** 10.3389/fcimb.2021.790243

**Published:** 2022-01-13

**Authors:** Wen-ying Long, Guo-hua Zhao, Yao Wu

**Affiliations:** ^1^ Central Laboratory, The Fourth Affiliated Hospital, Zhejiang University School of Medicine, Yiwu, China; ^2^ Neurology Department, The Fourth Affiliated Hospital, Zhejiang University School of Medicine, Yiwu, China

**Keywords:** atlastin, endoplasmic reticulum, KSHV, lytic activation, ER stress

## Abstract

Kaposi’s sarcoma-associated herpesvirus (KSHV) has two life cycle modes: the latent and lytic phases. The endoplasmic reticulum (ER) is the site for KSHV production. Furthermore, ER stress can trigger reactivation of KSHV. Little is known about the nature of the ER factors that regulate KSHV replication. Atlastin proteins (ATLs which include ATL1, ATL2, and ATL3) are large dynamin-related GTPases that control the structure and the dynamics of the ER membrane. Here, we show that ATLs can regulate KSHV lytic activation and infection. Overexpression of ATLs enhances KSHV lytic activation, whereas ATLs silence inhibits it. Intriguingly, we find that silencing of ATLs impairs the response of cells to ER stress, and ER stress can promote the lytic activation of KSHV. Our study establishes that ATLs plays a critically regulatory role in KSHV infection, thus expanding the known scope of biological processes controlled by ATLs to include KSHV infection.

## Introduction

Kaposi’s sarcoma-associated herpesvirus (KSHV) or human herpesvirus 8 (HHV8), is etiologically associated with KS, an angioproliferative malignancy of the human skin, and also with two angiolymphoproliferative disorders: body cavity-based B cell lymphoma (BCBL) (or primary effusion lymphoma [PEL]) and some forms of polyclonal B-cell proliferative multicentric Castleman’s disease (MCD) (Lange and Damania, 2020; Naipauer et al., 2020; Vega et al., 2020). An intensive research effort led to important progress regarding KSHV epidemiology, diagnosis, and potential therapeutic and prophylactic strategies (Gong et al., 2019; Holmes et al., 2020). The mechanisms involved in KSHV replication, host responses, and pathogenesis have also been extensively studied (Blumenthal et al., 2020; Yu et al., 2020; Zhao et al., 2020). However, the role of cellular proteins, in particular those present in the endoplasmic reticulum (ER), where viral production occurs, remains only partly characterized.

Like other herpesviruses, the replicative cycle of KSHV exists as latency and lytic replication. The transition between these states allows the establishment of a lifelong persistent infection, dissemination to sites of disease, and the spread to new hosts (Li et al., 2018; Ye et al., 2019; Juillard et al., 2020). KSHV mostly persists in the latent state during which it has a restricted latent gene expression program but can be reactivated and transitioned to the lytic state when triggered by stress conditions such as hypoxia or HIV coinfection (Gruffaz et al., 2020), or stimulated by other chemical signals such as 12-O-tetradecanoylphorbol-13-acetate (TPA), sodium butyrate (NaB), and valproate (VPA) (Deng et al., 2007; Bellare and Ganem, 2009; Purushothaman et al., 2015; Gonnella et al., 2017). Furthermore, ER stress can trigger lytic reactivation of KSHV (Johnston et al., 2019). Lytic reactivation in response to ER stress is primarily due to XBP1(s). The ER stress-sensing mechanism involves the presence of XBP1(s) target sequences in the promoters of immediate early viral genes (Johnston et al., 2019). KSHV expresses the immediate early protein replication and transcriptional activator (RTA), which is essential and sufficient to induce lytic replication (Broussard and Damania, 2020). The RTA promoter in KSHV contains at least one XBP1s response element with an ACGT core motif.

Latency-associated viral proteins have been well characterized in transformation and tumourigenesis pathways (DiMaio et al., 2020); however, a number of studies have shown that abrogation of KSHV lytic gene expression impairs the oncogenesis of several cancers (Dai et al., 2018; Manners et al., 2018). Furthermore, several lytically expressed proteins have been functionally tethered to the angioproliferative and anti-apoptotic phenotypes of virus-infected cells. As a result, the investigation and therapeutic targeting of KSHV lytic cycles may be essential for the treatment of their associated malignancies (Chen et al., 2019; Gabaev et al., 2020).

The ER is the largest cellular organelle and is involved in many processes, namely, protein production and degradation, cell signaling, and the synthesis and distribution of lipids. Several proteins that shape the ER have been identified, namely, the membrane-bending reticulon (RTN) and eceptor expression enhancing protein (REEP) and also atlastins (ATLs) and Lunapark (Chen et al., 2013). ATLs are large dynamin-related GTPases that dimerize in cis and trans to allow fusion of adjacent ER membranes. Humans have three ATLs (ATL1, ATL2, and ATL3), with redundant activities and various levels of expression in different cell types. Mutations in ATLs are associated with neurological diseases, such as hereditary sensory neuropathy and spastic paraplegia, and are characterized by axon and dendrite growth deficits (Fink, 2013). Therefore, ATLs are key cellular factors in regulating ER function, and important factors in human disease.

ATLs enhance ZIKV replication and cytopathic effects (Neufeldt et al., 2019). But the role of ATLs in KSHV is uncharacterized. Here, we report that ATLs enhance KSHV replication and silencing of ATLs impairs KSHV infection. We further characterize the underlying viral and cellular mechanisms and report that ATLs affect KSHV activation by regulating ER stress. Beyond adding KSHV infection to the scope of biological processes regulated by ATLs, our study provides insights about potential therapeutic targets against KSHV infection and KSHV-related malignancies.

## Materials and Methods

### Reagents

VPA, NaB, TPA, and tetracycline were purchased from Sigma-Aldrich Chemical Co. BFA was dissolved in DMSO at 1 M as a stock solution. VPA and NaB were dissolved in sterile ddH_2_O at 1 M as a stock solution. TPA was dissolved at 200 μg/ml concentration with sterile ddH_2_O. Tetracycline was dissolved in DMSO at 1 mg/ml as a stock solution.

### Cell Culture and Chemical Treatment

iSLK.rKSHV.219 cells and HUVEC cells were cultured in DMEM medium (Gibco), and the body cavity-based KSHV+ lymphoma cell line BCBL-1 was maintained in RPMI1640 medium (Gibco). All these cultures were supplemented with 10% fetal bovine serum (FBS) (Gibco) and 1% penicillin–streptomycin (Gibco).

iSLK.rKSHV.219 cells were treated with 1 μg/ml tetracycline plus 1 mM valproate (VPA) (Sigma) for 48 h to activate lytic replication. The BCBL-1 cells were subcultured at 3 ∗ 10^5^ cells/ml and treated with 20 ng/ml of 12-O-tetradecanoylphorbol-13- acetate (TPA) (Sigma) plus 0.3 mM NaB (Sigma) for 48 h to activate lytic replication. iSLK.rKSHV.219 cells were treated with 5 µg/ml brefeldin A (BFA) to induce ER stress.

### Cell Transfection

iSLK.rKSHV.219, HUVEC, and BCBL-1 cells were transfected with Lipofectamine ™ LTX (Invitrogen).

Specific siRNA oligonucleotides (GENERAL BIOSYSTEMS) for ATL1/2/3#1 were targeted against the following sequences: siATL1^#1^: CAA UAA ACC UGA UGG UAA ATT; siATL2^#1^: GGA GCU AUC CUU AUG AAC AUU CAU A; siATL3^#1^: GCC CUG ACU UUG AUG GGA AAU UAA A. Specific siRNA oligonucleotides (Invitrogen) for ATL1/2/3#2 were targeted against the following sequences: siATL1^#2^: UUU ACC AUC AGG UUU AUU GTT; siATL2^#2^: UCC UGG UCU UAA AGU UGC AAC UAA U; siATL3^#2^: GGG CUA CAU CAG GUA UUC UGG UCA A. Specific siRNA oligonucleotides (GENERAL BIOSYSTEMS) for CHOP were targeted against the following sequences: CHOP^#1^: GCC UAG GUC UCU UAG AUG A, CHOP^#2^: GAA CUA GGA AAC GGA AAC A. Specific siRNA oligonucleotides (GENERAL BIOSYSTEMS) for BIP were targeted against the following sequences: BIP^#1^:UAG GGU GUG UGU UCA CCU UCA, BIP^#2^: GGA GCG CAU UGA UAC UAG A. Specific siRNA oligonucleotides (GENERAL BIOSYSTEMS) for RTN2 were targeted against the following sequences: RTN2^#1^: GUU CCA AUU UUG GAA UUG UCC, RTN2^#2^: CCG AUA UGG GGA GUA AAG UGG. Specific siRNA oligonucleotides (GENERAL BIOSYSTEMS) for REEP2 were targeted against the following sequences: REEP2^#1^: CAA AAA ACG UGA AGG AAU AUG, REEP2^#2^: CAC AUG UUC CCA CAU UAA AAA.

### Infection of HUVEC Cells With KSHV

HUVEC cells were plated on 6-well plates at 5 × 10^5^ cells/ml. The next day, cells were inoculated for 4 h at 37°C with 1.5 ml of cell culture media derived from iSLK.rKSHV.219 cells that have been treated with tetracycline plus VPA. This was followed by replacing with fresh DMEM media and cells were incubated at 37°C and 5% CO2 for 48 h.

### Quantitative Reverse Transcription-PCR (qRT-PCR)

Total RNA was extracted from cells using Trizol (life technologies) according to the manufacturer’s protocol. RNA was converted to cDNA by using RevertAid First Strand cDNA Synthesis Kit (Thermo) according to the manufacturer’s protocol.

Relative transcript levels of selected cellular and genes were determined with gene-specific primers plus SYBR^®^ Premix Ex Taq™II (Tli RNaseH Plus) (TaKaRa) by 7500 fast real-time PCR system (Applied Biosystems). The sequences of the primers used are shown in **Table 1**.

**Table 1:** Oligonucleotides used for PCR and qRT-PCR analyses.

Gene	Forward	Reverse
ORF50	TATCCAGGAAGCGGTCTCAT	GGGTTAAAGGGGATGATGCT
actin	GGGAAATCGTGCGTGACAT	GTCAGGCAGCTCGTAGCTCTT
ORFK8.1	TGGTCGGCGGTTCAGTCATCAA	GCGGCCGCTAAGAAAATCGA
ORF59	TTAGAAGTGGAAGGTGTGCC	TCCTGGAGTCCGGTATAGAATC
ORF9	TAGGCGCTTCGTGCTGG	CCGGATTGCTGCACTCGTA
FAM105B	GACAGCTTCTGAGGAACCACCT	TCCGTGTTGTACTTGGAGAGCC
vLANA	TCCAAAGTGTCAATGGAAGT	GTAGATGGGTCGTGAGAACA
vCyclin	AACACCCTGATTACAAAAGC	ATCAAAGTCCGAAACAGATG
hXBP1s	AACCAGGAGTTAAGACAGCGCTT	TCGTCAGCATGAACTTGAGAG
BIP	GGCGTGGTAGTGCAAGCTGA	CCTATCCTTGGGCAGTATTGGATTC
CHOP	GCGCATGAAGGAGAAAGAAC	TCACAATTCGGTCAATCAGA
ATL1	CAGCACCTCCAGCTTTTCACTG	CACCACCATCGGCTCCATATGA
ATL2	TCCTTTTGCCACATCCTGGTCT	GCAAGCAGCAATGGAACCAGAT
ATL3	AAGATCTGCCTCACCCCAAGTC	CTCCCCCACAAACCTCTTCCAT
RTN2	ATTCACCATCCCCCTGCTGTAC	AACTGATTGGTCACCAACCCCA
REEP2	TGCTCATCTTTGGCACCC TGTA	CCGTGGTGAAGAAGGCAAAGAC

Relative expression levels were calculated using the ΔΔCT method after normalization to actin. Individual samples were assayed in duplication.

### Quantitative Analysis of KSHV Virions in Supernatant

Viral DNA was prepared from culture media by using the AxyPrep TM Body Fluid Viral DNA/RNA Miniprep Kit (AXYGEN) according to the manufacturer’s protocol. A fixed amount of plasmid FAM105B was added to the media before DNA extraction, to serve as a normalization control during qPCR step. Extracted DNA was used to amplify the KSHV LANA gene by qPCR using the following primers: LANA-F, 5’-TCC AAA GTG TCA ATG GAA GT, and LANA-R, 5’-GTA GAT GGG TCG TGA GAA CA. Relative expression levels were calculated using the ΔΔCT method after normalization to FAM105B (FAM105B plasmid was added to supernatant as a control). Individual samples were assayed in triplicates.

## Results

### ATLs Overexpression Promotes Spontaneous KSHV Lytic Reactivation in iSLK.rKSHV.219 Cells

**Figure 1:**
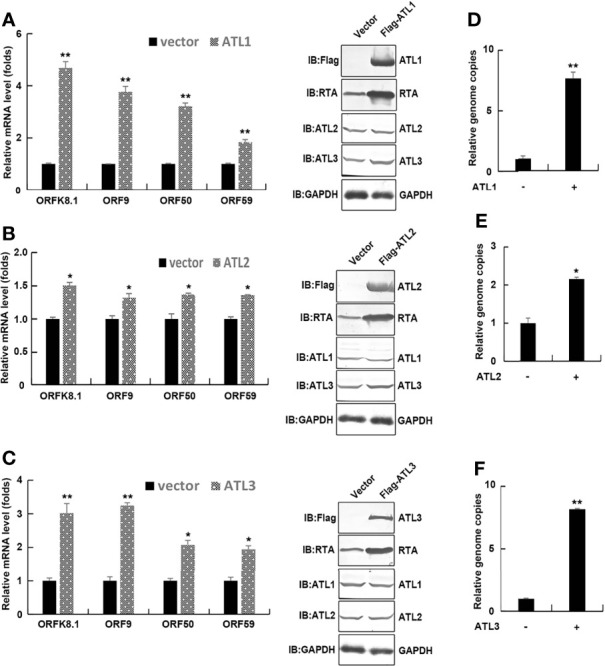
ATLs overexpression promotes spontaneous KSHV lytic reactivation in iSLK. rKSHV.219 cells. iSLK.rKSHV.219 cells were transfected with vector, ATL1, ATL2 or ATL3 respectively. Forty eight hours later, mRNA levels of KSHV lytic genes *ORFK8.1*, *ORF9*, *ORF50*, and *ORF59* were measured by RT-qPCR, with normalization to actin using the ΔΔCT method. Proteins were detected by western blot with indicated antibodies **(A–C)**. Viral DNA in the media was quantified using qPCR, with normalization to an added plasmid FAM105B using the ΔΔCT method **(D–F)**. Data are presented as means of three technical replicates (n = 3, group values are indicated by mean ± SEM; *p <0.05; **p <0.01).

Seeking host cellular factors that may restrict KSHV lytic reactivation, and with particular interest in ER, we initially focused on the ER-shaping proteins. We chose the iSLK.rKSHV.219 cell line that harbors recombinant KSHV (rKSHV.219) with constitutive GFP expression indicating latent infection and RFP expression reporting lytic reactivation upon lytic cycle induction as our experimental system. Following pilot experiments with the cell line, we focused on the ER related proteins-ATLs. And we firstly determined the physiological expression of the three proteins in their model cells (iSLK.rKSHV.219 and BCBL) (**Figure S1A**).To determine whether ATLs functions in KSHV lytic replication, we transfected iSLK.rKSHV.219 cells with an empty vector, ATL1, ATL2 or ATL3 respectively. After 48 h, we detected the expression of KSHV lytic gene by RT-PCR. The results showed that, compared to empty vector-transfected iSLK.rKSHV.219 cells, ATL1 (**Figure 1A**), ATL2 (**Figure 1B**) or ATL3 (**Figure 1C**)-transfected cells had significant increases in the mRNA levels of representative KSHV lytic genes *ORFK8.1*, *ORF9*, *ORF50*, and *ORF59*. RTA is a lytic protein encoded by KSHV *ORF50*. The expression level of RTA detected by western blot was consistent with that of mRNA. Meanwhile, ATL overexpression resulted in an increase in viral particles in the cell media (**Figures 1D–F**). Furthermore, we have examined whether the increasing expression of any one of the three ATL proteins would affect the expression of other two ATL proteins in iSLK.rKSHV.219 cells. The results showed that the expression of single ATL had no significant effect on the expression of other two ATL proteins (**Figures S1B–D**). These results indicate that ATLs overexpression promotes spontaneous KSHV lytic reactivation in iSLK.rKSHV.219 cells.

### ATLs Overexpression Promotes VPA Induced KSHV Lytic Reactivation in iSLK.rKSHV.219 Cells

**Figure 2:**
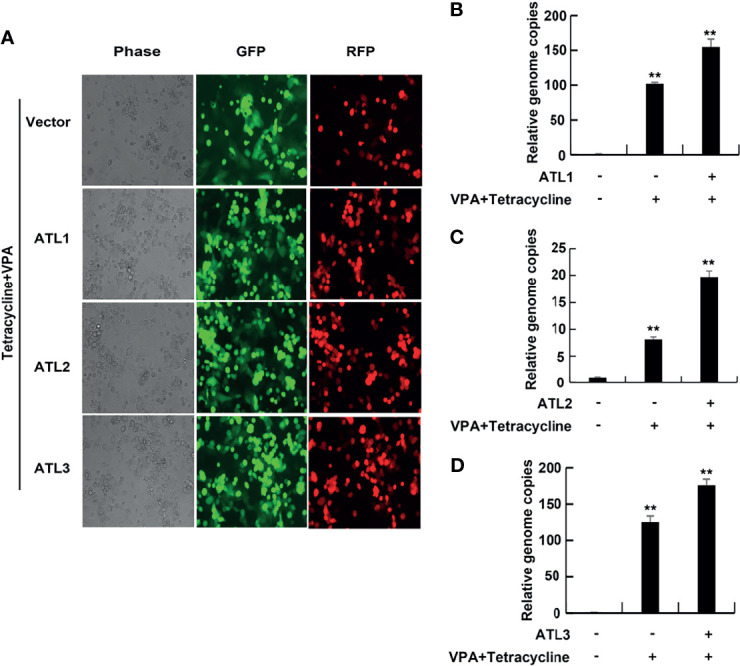
ATLs overexpression promotes VPA induced KSHV lytic reactivation and virus particle production in iSLK.rKSHV.219 cells. iSLK.rKSHV.219 cells were transfected with vector, ATL1, ATL2 or ATL3 respectively followed by treatment with tetracycline plus valproate (VPA) for 48 hours to induce KSHV lytic reactivation. **(A)** The cells were photographed for GFP and RFP fluorescence. **(B-D)** Viral DNA in the media was quantified using qPCR, with normalization to an added plasmid FAM105B using the ΔΔCT method. Data are presented as means of three technical replicates (n=3, group values are indicated by mean ± SEM; ** p<0.01).

Most of KSHV infected cells are in latent state. Treatment with VPA can induce KSHV to enter the lytic phase, so as to better study the effect of ATL on the lytic reactivation of KSHV. To determine the role of ATLs in VPA induced KSHV lytic replication, we transfected iSLK.rKSHV.219 cells with an empty vector, ATL1, ATL2 or ATL3 respectively. Upon treatment with tetracycline and valproate (VPA) to induce lytic reactivation, photomicrographs revealed more significant number of RFP + cells in ATLs-transfected cells than empty vector-transfected cells (**Figure 2A**). Consistently, there were more viral particles in media from ATLs-transfected cells than from empty vector-transfected cells (**Figures 2B–D**). In addition, we detected the expression of two representative KSHV latency genes *vCyclin* and *vLANA* related to KSHV DNA replication in host cells. The results showed that overexpression of ATL proteins could increase the expression levels of *vCyclin* (**Figure S2A**) and *vLANA* (**Figure S2B**), and knockdown of ATL proteins could inhibit the expression levels of *vCyclin* (**Figure S2C**) and *vLANA* (**Figure S2D**), which was consistent with the situation of viral DNA in the media.

**Figure 3:**
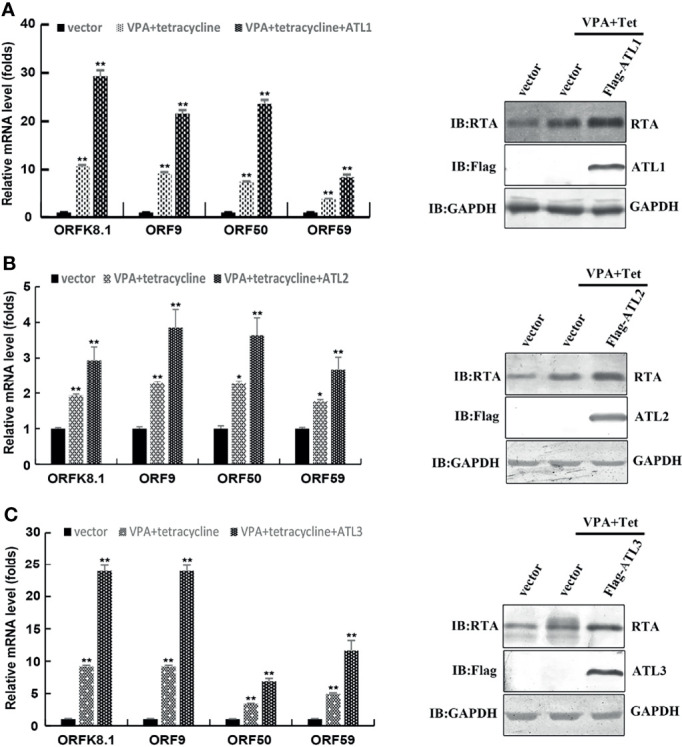
ATLs overexpression promotes VPA induced expression of KSHV lytic genes in iSLK.rKSHV.219 cells. iSLK.rKSHV.219 cells were transfected with vector, ATL1, ATL2 or ATL3 respectively followed by treatment with tetracycline plus valproate (VPA) for 48 h to induce KSHV lytic reactivation. The effect of ATL1 **(A)**, ATL2 **(B)**, and ATL3 **(C)** on mRNA levels of KSHV lytic genes *ORFK8.1*, *ORF9*, *ORF50*, and *ORF59* were measured by RT-qPCR, with normalization to actin using the ΔΔCT method. Data are presented as means of three technical replicates (n=3, group values are indicated by mean ± SEM; *p<0.05; **p<0.01). Proteins were detected by western blot with indicated antibodies.

Furthermore, RT-qPCR showed that, compared to empty vector-transfected iSLK. rKSHV.219 cells, ATLs-transfected cells had significant increases in the mRNA levels of representative KSHV lytic genes *ORFK8.1*, *ORF9*, *ORF50*, and *ORF59* and the expression level of RTA detected by western blot was consistent with that of mRNA (**Figures 3A–C**). These results indicate that ATL overexpression promotes VPA induced KSHV lytic reactivation in iSLK.rKSHV.219 cells.

### Silencing of ATLs Impairs Spontaneous KSHV Lytic Reactivation in iSLK.rKSHV.219 Cells

**Figure 4:**
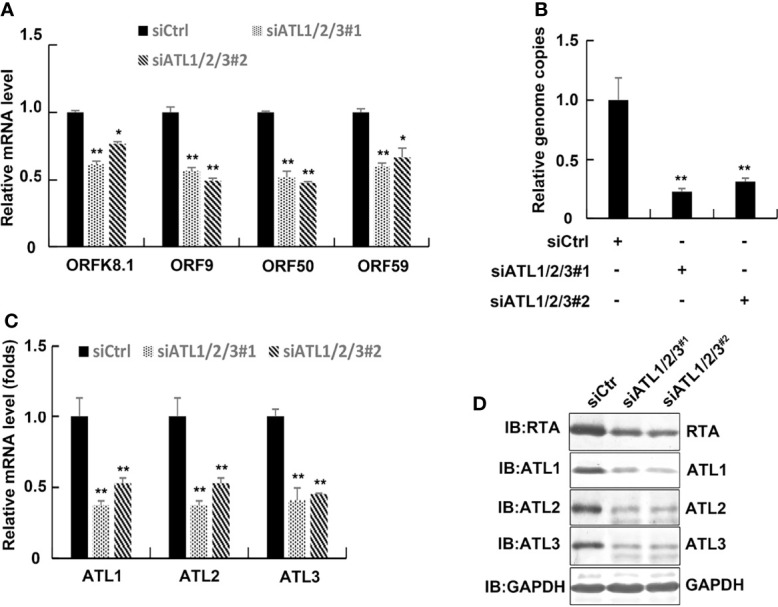
Silencing of ATLs impairs spontaneous KSHV lytic reactivation in iSLK.rKSHV.219 cells. iSLK.rKSHV.219 cells were transfected with siCtrl, siATL1/2/3#1 or siATL1/2/3#2 respectively. Forty eight hours later, mRNA levels of KSHV lytic genes *ORFK8.1*, *ORF9*, *ORF50*, and *ORF59* were measured by RT-qPCR, with normalization to actin using the ΔΔCT method **(A)**. Viral DNA in the media was quantified using qPCR, with normalization to an added plasmid FAM105B using the ΔΔCT method **(B)**. Knockdown efficiencies of siATL1/2/3 in iSLK.rKSHV.219 cells were measured by RT-qPCR **(C)**. Data are presented as means of three technical replicates (n = 3, group values are indicated by mean ± SEM; *p <0.05; **p <0.01). **(D)** Proteins were detected by western blot with indicated antibodies.

We next used siRNAs to knock down ATLs expression in iSLK.rKSHV.219 cells. The results of RT-PCR showed that compared to control siRNA-treated cells, introduction of 2 different siRNA pairs targeting ATL1/2/3 all resulted in significant decreases in the mRNA levels of representative KSHV lytic genes *ORFK8.1*, *ORF9*, *ORF50*, and *ORF59* (**Figure 4A**). Meanwhile, silencing of ATLs resulted in a decrease in viral particles in the cell media (**Figure 4B**). The efficiency of ATLs silence was verified by RT-qPCR (**Figure 4C**) and the expression level of RTA detected by western blot was consistent with that of mRNA (**Figure 4D**). These results indicate that silencing of ATLs impairs spontaneous KSHV lytic reactivation in iSLK.rKSHV.219 cells.

### Silencing of ATLs Impairs VPA Induced KSHV Lytic Reactivation and Virus Particle Production in iSLK.rKSHV.219 Cells

**Figure 5:**
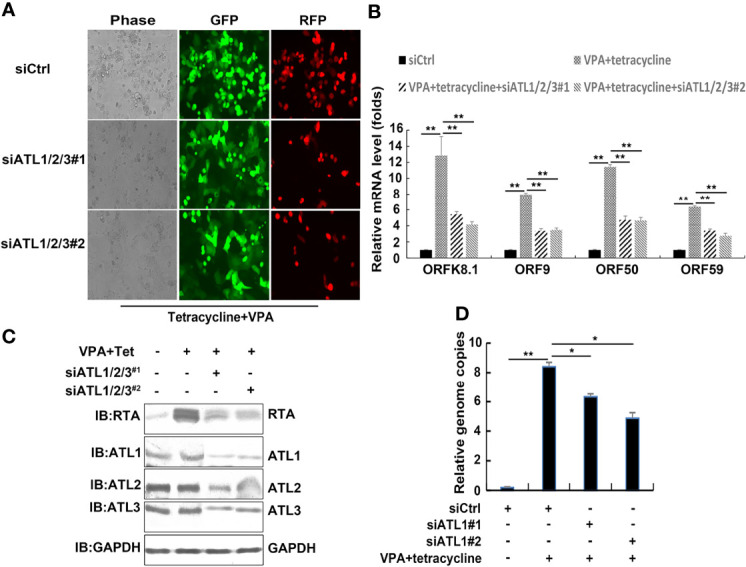
Silencing of ATLs impairs VPA induced KSHV lytic reactivation and virus particle production in iSLK.rKSHV.219 cells. iSLK.rKSHV.219 cells were transfected with siCtrl, siATL1/2/3#1 or siATL1/2/3#2 respectively followed by treatment with tetracycline plus valproate (VPA) for 48 h to induce KSHV lytic reactivation. **(A)** The cells were photographed for GFP and RFP fluorescence. **(B)** mRNA levels of KSHV lytic genes *ORFK8.1*, *ORF9*, *ORF50*, and *ORF59* were measured by RT-qPCR, with normalization to actin using the ΔΔCT method. **(C)** Proteins were detected by western blot with indicated antibodies. **(D)** Viral DNA in the media was quantified using qPCR, with normalization to an added plasmid FAM105B using the ΔΔCT method. Data are presented as means of three technical replicates (n = 3, group values are indicated by mean ± SEM; *p <0.05; **p <0.01).

To determine silencing of ATLs whether functions in VPA induced KSHV lytic replication, we transfected iSLK.rKSHV.219 cells with control siRNA or 2 different siRNA pairs targeting ATL1/2/3. Upon treatment with tetracycline and VPA to induce lytic reactivation, photomicrographs revealed less significant number of RFP + cells in siATL1/2/3-transfected cells than siCtrl-transfected cells (**Figure 5A**). Silencing of ATLs reduced mRNA levels of representative KSHV lytic genes *ORFK8.1*, *ORF9*, *ORF50*, and *ORF59* (**Figure 5B**) and the expression level of RTA detected by western blot was consistent with that of mRNA (**Figure 5C**). Consistently, there were less viral particles in media from siATL1/2/3-transfected cells than from siCtrl-transfected cells (**Figure 5D**). Furthermore, we examined the effect of single ATL silencing on spontaneous (**Figures S3A, B**) or VPA induced lytic reactivation of KSHV (**Figures S3C, D**). The knockdown efficiency was ensured by RT-PCR (**Figures S3E, F**). The results showed that knockdown of single ATL protein could also inhibit the lytic reactivation of KSHV, but its inhibitory effect was significantly weaker than that of knockdown of three ATL proteins at the same time. We also rescued ATL1 (**Figure S4A**), ATL2 (**Figure S4B**), or ATL3 (**Figure S4C**) in our ATL1/2/3 knockdown experiments and the rescue efficiency was ensured by RT-PCR (**Figures S4E, F**). The experimental results show that the replenishment of single ATL protein can restore the lytic reactivation of KSHV to a great extent. These results show that silencing of ATLs impairs VPA induced KSHV lytic reactivation and virus particle production in iSLK.rKSHV.219 cells and ATL proteins may have a synergistic effect in regulating the lytic reactivation of KSHV.

### Silencing of ATLs Impairs Spontaneous and TPA Induced KSHV Lytic Reactivation and Virus Particle Production in BCBL-1 Cells

**Figure 6:**
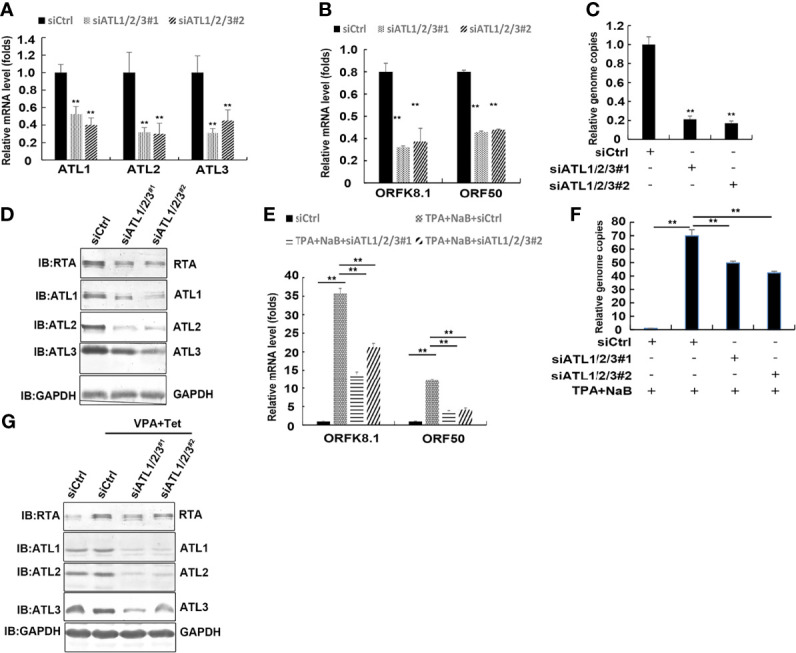
Silencing of ATLs impairs spontaneous and TPA induced KSHV lytic activation and virus particle production in BCBL-1 cells. BCBL-1 cells were transfected with siCtrl, siATL1/2/3#1 or siATL1/2/3#2 respectively. After 48 hours, knockdown efficiencies of siATL1/2/3 in BCBL-1 cells were measured by RT-qPCR **(A)**. mRNA levels of KSHV lytic genes ORFK8.1 and ORF50 were measured by RT-qPCR, with normalization to actin using the ΔΔCT method **(B)**. Viral DNA in the media was quantified using qPCR, with normalization to an added plasmid FAM105B using the ΔΔCT method **(C)**. BCBL-1 cells were transfected with siCtrl, siATL1/2/3#1 or siATL1/2/3#2 respectively followed by treatment with TPA plus NaB for 48 hours to induce KSHV lytic reactivation. **(D)** Proteins were detected by western blot with indicated antibodies. **(E)** mRNA levels of KSHV lytic genes ORFK8.1 and ORF50 were measured by RT-qPCR, with normalization to actin using the ΔΔCT method. **(F)** Viral DNA in the media was quantified using qPCR, with normalization to an added plasmid FAM105B using the ΔΔCT method. Data are presented as means of three technical replicates (n=3, group values are indicated by mean ± SEM; ** p<0.01). **(G)** Proteins were detected by western blot with indicated antibodies.

To determine whether ATLs functions in viral reactivation in other KSHV-infected cells, we introduced siRNAs targeting ATL1/2/3 into BCBL-1 cells to greatly deplete ATLs mRNA levels (**Figure 6A**). BCBL-1 harbors latently-infected KSHV and is a B cell lymphoma cell line derived from the peritoneal effusion of a patient with primary effusion lymphoma. Silencing of ATLs impairs spontaneous KSHV lytic activation. There were decreased mRNA levels of representative KSHV lytic genes *ORFK8.1* and *ORF50* (**Figure 6B**). Viral particles in media were also reduced (**Figure 6C**) and the expression level of RTA detected by western blot was consistent with that of mRNA (**Figure 6D**).

Furthermore, the ATLs-silenced BCBL-1 cells were treated with NaB plus TPA induce KSHV lytic reactivation. RT-qPCR revealed that the mRNA levels of the lytic genes *ORFK8.1* and *ORF50* were significantly decreased in ATLs-silenced cells (**Figure 6E**). Consistently, there were less viral particles in media from siATL1/2/3-transfected cells than from siCtrl-transfected cells (**Figure 6F**). The protein expression of RTA was consistent with its mRNA expression (**Figure 6G**). These results indicate that silencing of ATLs impairs spontaneous and TPA induced KSHV lytic activation and virus particle production in BCBL-1 cells.

### ATL Proteins Have Certain Specificity in the Regulation of KSHV Lytic Reactivation

**Figure 7:**
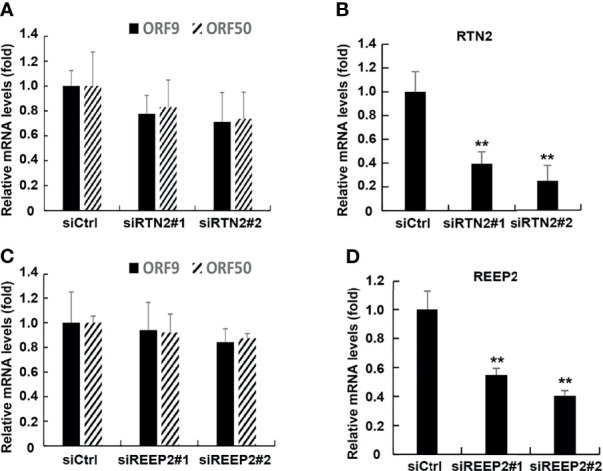
ATL proteins have certain specificity in the regulation of KSHV lytic reactivation. The knockdown of RTN2 had no significant effect on the expression of KSHV lytic genes. **(A)** iSLK.rKSHV.219 cells were transfected with siCtrl, siRTN2^#1^, or siRTN2^#2^ respectively. 48 hours later, mRNA levels of KSHV lytic genes ORF9 and ORF50 were measured by RT-qPCR, with normalization to actin using the ∆∆CT method. **(B)** Knockdown efficiencies of siRTN2 in iSLK.rKSHV.219 cells were measured by RT-qPCR. Data are presented as means of three technical replicates (n=3, group values are indicated by mean ± SEM; *p < 0.05; **p < 0.01). The knockdown of REEP2 had no significant effect on the expression of KSHV lytic genes. **(C)** iSLK.rKSHV.219 cells were transfected with siCtrl, siREEP2^#1^, or siREEP2^#2^ respectively. 48 hours later, mRNA levels of KSHV lytic genes ORF9 and ORF50 were measured by RT-qPCR, with normalization to actin using the ΔΔCT method. **(D)** Knockdown efficiencies of siREEP2 in iSLK.rKSHV.219 cells were measured by RT-qPCR. Data are presented as means of three technical replicates (n=3, group values are indicated by mean ± SEM; **p < 0.01).

To argue for the specific effects of ATLs, we examined the effect of other ER-forming proteins, namely, RTN2 and REEP2, on lytic reactivation of KSHV. After knocking down RTN2 in iSLK.rKSHV.219 cells, the expression level of KSHV lytic genes was not significantly affected (**Figure 7A**). The efficiency of RTN2 silence was verified by RT-qPCR (**Figure 7B**). In additional, the results of RT-PCR also showed that knockdown of REEP2 did not significantly affect the lytic reactivation of KSHV (**Figure 7C**). And the efficiency of ATLs silence was verified by RT-qPCR (**Figure 7D**).

In summary, ATL proteins have certain specificity in the regulation of KSHV lytic reactivation.

### Silencing of ATLs Impairs KSHV *De Novo* Infection in HUVEC Cells

**Figure 8:**
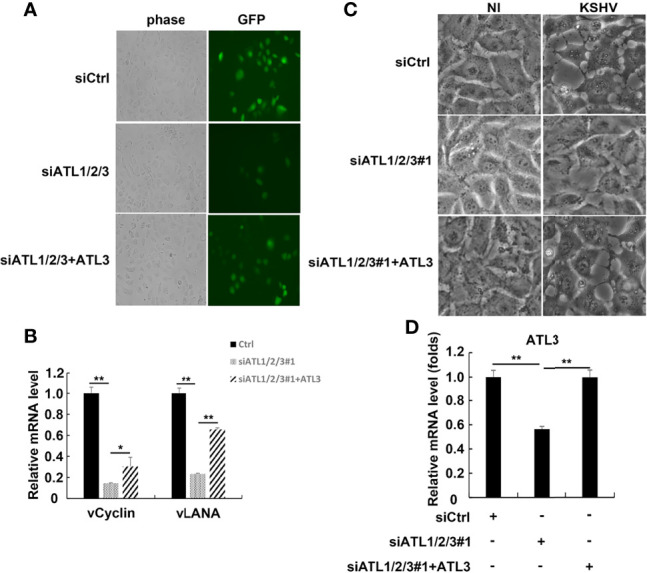
Silencing of ATL impairs KSHV *de novo* infection in HUVEC cells. HUVEC cells were transfected with siCtrl, siATL1/2/3#1 or siATL1/2/3#1 plus ATL3 respectively followed by infection with rKSHV219 for 24 h **(A)** The cells were photographed for GFP fluorescence. **(B)** mRNA levels of KSHV latent genes *vCyclin* and *vLANA* were measured by RT-qPCR, with normalization to actin using the ΔΔCT method. **(C)** Virus-induced vacuoles were observed by light microscopy. **(D)** Knockdown efficiency of siATL1/2/3 and replenishment efficiency of ATL3 in HUVEC cells were measured by RT-qPCR. Data are presented as means of three technical replicates (n=3, group values are indicated by mean ± SEM; *p < 0.05; **p < 0.01).

We also speculated that ATLs may regulate *de novo* KSHV infection. To test this idea, we introduced siRNAs targeting ATLs into HUVEC cells to generate ATLs-silenced cell lines with greatly depleted ATLs. Then, we transfected ATLs-silenced HUVEC cells with empty vector or ATL3. After transfection, we infected these cell lines with rKSHV.219 and found that, compared to control or ATL3-replenishment cells, ATLs silence resulted in a significant decrease in the proportion of GFP + cells (**Figure 8A**). Furthermore, this decrease corresponded to the decrease in the mRNA levels of KSHV genes *vLANA* and *vCyclin* (**Figure 8B**). Interestingly, large ER-derived cytoplasmic vacuoles appeared in KSHV infected cells. In ATLs-silenced KSHV-infected HUVEC cells, the formation of these vacuoles was strongly decreased at 24 h. Replenishment of ATL3 increased virus-induced vacuoles in ATL-silenced KSHV-infected HUVEC cells (**Figure 8C**). The efficiency of ATLs silence was verified by RT-qPCR (**Figure 8D**). These results indicate that silencing of ATL impairs KSHV *de novo* infection in HUVEC cells.

### Silencing of ATLs Impairs the Response of Cells to ER Stress, and ER Stress can Promote the Lytic Reactivation of KSHV

**Figure 9:**
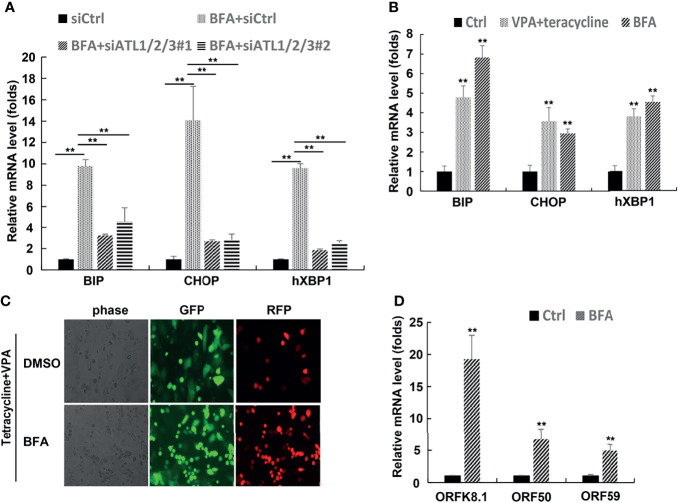
Silencing of ATLs impairs the response of cells to endoplasmic reticulum (ER) stress, and ER stress can promote the lytic reactivation of KSHV. **(A)** iSLK.rKSHV.219 cells were transfected with siCtrl, siATL1/2/3#1 or siATL1/2/3#1 respectively followed by treated with BFA for 24 hours. mRNA levels of ER stress related genes *BIP, CHOP* and *hXBP1(s)* were measured by RT-qPCR, with normalization to actin using the ΔΔCT method. **(B)** iSLK.rKSHV.219 cells were treated with tetracycline plus VPA or BFA for 24 hours, mRNA levels of ER stress related genes *BIP, CHOP* and *hXBP1(s)* were measured by RT-qPCR, with normalization to actin using the ΔΔCT method. iSLK.rKSHV.219 cells were treated with DMSO or BFA for 1 hours followed by treatment with tetracycline plus VPA for 48 hours to induce KSHV lytic reactivation. The cells were photographed for GFP and RFP fluorescence **(C)**. mRNA levels of KSHV lytic genes ORFK8.1, ORF50 and ORF59 were measured by RT-qPCR **(C)**. Data are presented as means of three technical replicates (n=3, group values are indicated by mean ± SEM; ** p < 0.01).

ATLs are large dynamin-related GTPase that dimerize in cis and trans to allow fusion of adjacent ER membrances. ER stress can trigger reactivation of EBV, KSHV, and MHV68. In order to find out whether the lytic activation of KSHV caused by ATLs is related to ER stress, we first detected the effect of ATLs silence on ER stress. The results of RT-PCR showed that mRNA levels of ER stress related genes *BIP*, *CHOP*, and *hXBP1(s)* were decreased in BFA-treated ATLs-silenced iSLK.rKSHV219 cells when compared with control cells (**Figure 9A**). In other words, silencing of ATLs impairs the response of cells to ER stress. Furthermore, treating iSLK.rKSHV219 cells with VPA or brefeldin A (BFA) can increase mRNA levels of ER stress related genes *BIP*, *CHOP*, and *hXBP1(s)* (**Figure 9B**). Then we treated iSLK.rKSHV219 cells with DMSO or BFA for 24 h. Photomicrographs revealed more significant number of RFP + cells in BFA-treated cells than DMSO-treated cells (**Figure 9C**). Consistently, there were increased mRNA levels of representative KSHV lytic genes *ORFK8.1*, *ORF9*, *ORF50*, and *ORF59 in* BFA-treated cells (**Figure 9D**).

### Knockdown of CHOP or BIP can Antagonize the Promoting Effect of ATL Proteins on the Lytic Reactivation of KSHV to a Great Extent

**Figure 10:**
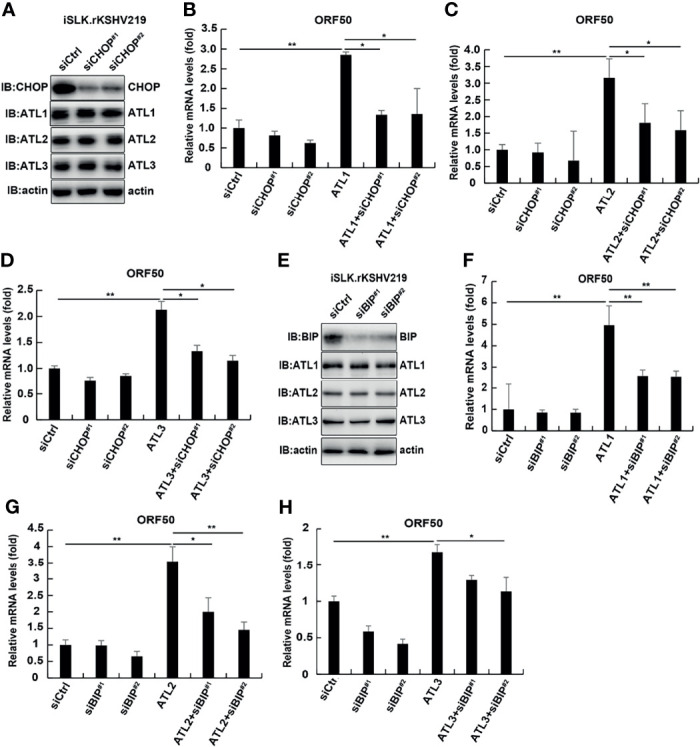
Knockdown of CHOP and BIP can antagonize the promoting effect of ATL proteins on the lytic reactivation of KSHV to a great extent. **(A)** iSLK.rKSHV.219 cells were transfected with siCtrl, siCHOP^#1^, or siCHOP^#2^ respectively. Forty eight hours later, protein levels of BIP, ATL1, ATL2, and ATL3 were measured by western blot with indicated antibody. **(B–D)** iSLK.rKSHV.219 cells were transfected with siCtrl, siCHOP^#1^, or siCHOP^#2^ respectively. Six hours later, cells were transfected with ATL1, ATL2, or ATL3 respectively. Forty eight hours later, mRNA levels of KSHV lytic genes *ORF50* were measured by RT-qPCR, with normalization to *actin* using the ΔΔCT method. Data are presented as means of three technical replicates (n = 3, group values are indicated by mean ± SEM; *p <0.05; **p <0.01). **(E)** iSLK.rKSHV.219 cells were transfected with siCtrl, siBIP^#1^, or siBIP^#2^ respectively. Forty eight hours later, protein levels of BIP, ATL1, ATL2, and ATL3 were measured by western blot with indicated antibody. **(F–H)** iSLK.rKSHV.219 cells were transfected with siCtrl, siBIP^#1^, or siBIP^#2^ respectively. Six hours later, cells were transfected with ATL1, ATL2, or ATL3 respectively. Forty eight hours later, mRNA levels of KSHV lytic genes *ORF50* were measured by RT-qPCR, with normalization to *actin* using the ΔΔCT method. Data are presented as means of three technical replicates (n = 3, group values are indicated by mean ± SEM; *p <0.05; **p <0.01).

To further confirm whether ATL proteins had a direct effect on viral replication or an indirect effect by acting on the ER stress response. We examined the effect of CHOP or BIP knockdown on the expression of ATL proteins and the lytic reactivation of KSHV which was promoted by ATLs overexpression. The results showed that knockdown of CHOP did not affect the expression of ATL proteins (**Figure 10A**). In additional, compared with wild-type cells, overexpression of ATL1 (**Figure 10B**), ATL2 (**Figure 10C**), or ATL3 (**Figure 10D**) in CHOP knockdown cells significantly reduced the promotion of KSHV lytic reactivation.

Similarly, knockdown of BIP could not significantly affect the expression of ATL proteins in cells (**Figure 10E**). After knocking down BIP in iSLK.rKSHV.219 cells, ATL1 (**Figure 10F**), ATL2 (**Figure 10G**), or ATL3 (**Figure 10H**) was overexpressed, and its promoting effect on the lytic reactivation of KSHV was significantly weakened. That is, CHOP or BIP should be involved in the regulation of ATL proteins on KSHV lytic reactivation and ATL proteins have an indirect effect on KSHV lytic reactivation by acting on the ER stress response. In additional, we detected the effect of ATL1 (**Figure S5A**), ATL2 (**Figure S5B**), or ATL3 (**Figure S5C**) single depletion on BFA induced ER stress. The results showed that knockdown of single ATL protein had no significant effect on BFA induced ER stress. Moreover, ER stress had no obvious effect on the expression of ATL proteins (**Figures S5D**–**F**). This suggests that ATL proteins may also have a synergistic effect on the regulation of ER stress, which is consistent with the synergistic regulation of KSHV lytic reactivation. This further proves that ATL proteins affect the lytic reactivation of KSHV through the regulation of UPR.

## Discussion

The ER is the largest cellular organelle which links many cellular organelles and is involved in numerous processes. Several proteins that shape the ER have been identified, including ATLs, RTNs, REEPs, and Lunapark (Chen et al., 2013). Mutations in ATLs have been linked to neurodegenerative diseases, namely, hereditary spastic paraplegia (Fink, 2013). Furthermore, ATLs enhance ZIKV replication and cytopathic effects (Neufeldt et al., 2019). But the role of ATL proteins in KSHV is uncharacterized. In this study, we revealed that ATL overexpression promotes spontaneous and induced KSHV lytic reactivation in KSHV-harboring cells. Opposing this process, silencing of ATL impairs KSHV lytic reactivation. In the process of further exploring the mechanism of ATLs, we found that silencing of ATLs impairs the response of cells to ER stress, while ER stress can promote the lytic reactivation of KSHV. These data indicate that ATLs may be a potential treatment target for aggressive PEL and KSHV infection.

In order to ensure the smooth progress of the experiment, we first detected the expression of ATL proteins in iSLK.rKSHV.219 and BCBL-1 cells, and detected the effect on their mutual expression level (**Figure S1**). Subsequently, we have demonstrated that spontaneous KSHV lytic reactivation is promoted by ATLs in KSHV-harboring cells; consistently, lytic activation of KSHV is suppressed in ATLs-silence cells (**Figures 1**, **4**). Moreover, because a single ATL protein knockdown cannot inhibit the lytic reactivation of KSHV to a great extent (**Figure S3**), and supplementing a single ATL protein in ATL proteins knockout cells can significantly restore the lytic reactivation of KSHV (**Figure S4**), it can be inferred that several ATL proteins should have a synergistic effect on the regulation of KSHV lytic reactivation.

Although in our experiment, we detected that RTN2 and REEP2 had no significant effect on the lytic reactivation of KSHV (**Figure 7**), which could explain the specificity of ATL proteins on the regulation of KSHV to a certain extent. However, according to the existing studies, there are several proteins that shape the ER. So, it will be interesting to determine whether other ER related proteins have effects on KSHV lytic activation. In additional, as ATLs can enhance virus production, our next step will be to explore whether it also affects the infection efficiency of these viruses.

Since KSHV can also be activated by some stimuli such as hypoxia, HIV infection, chemical reagents VPA, NaB, and TPA (Manners et al., 2018), we also tested the effect of ATLs on KSHV lytic reactivation induced by VPA or TPA. The results showed that ATLs also enhanced lytic reactivation of KSHV induced by VPA or TPA (**Figures 2**–**6**). These results suggested that ATLs might affect a common event in KSHV reactivation or virion assembly processes induced by various stimuli. Moreover, other γ-herpesviruses also have lytic activation events similar to KSHV, it will be interesting to determine whether ATLs can modulate other γ-herpesviruses infection.

In our study, silencing of ATLs impaired KSHV *de novo* infection and virus-induced vacuoles (**Figure 8**). Many signaling pathways have been reported to be involved in the regulation of KSHV *de novo* infection, such as MAPK, NF-κB, antiviral and inflammation signaling pathways (Lee et al., 2016; Uppal et al., 2018; Wei and Lan, 2018; Zhao et al., 2018; Golas et al., 2020). Thus, it will be fascinating to determine whether ATLs have effects on a series of events involving growth and development, inflammation and antiviral.

We determined that silencing of ATLs impairs the response of cells to ER stress, and ER stress can promote the lytic reactivation of KSHV (**Figure 9**). Knockdown of CHOP or BIP can antagonize the promoting effect of ATL proteins on the lytic reactivation of KSHV to a great extent (**Figure 9**). From this we speculate that ATL proteins may have an indirect effect by acting on the ER stress response. Knockdown of a single ATL protein cannot significantly affect ER stress (**Figure S5**), so it suggests that ATL proteins may also have a synergistic effect on the regulation of ER stress, which is consistent with the synergistic regulation of KSHV lytic reactivation. These results further prove that ATL proteins affects the lytic reactivation of KSHV through the regulation of UPR. It has been reported that excessive ER stress can inhibit the lytic reactivation of KSHV, while an appropriate amount of ER stress can promote the lytic reactivation of KSHV (Johnston et al., 2019). ATLs may control the ER stress in KSHV-harboring cells at a level that can just promote the lytic reactivation of KSHV without causing it to be inhibited.

In conclusion, ATLs overexpression promotes while ATLs silently suppress KSHV replication and reactivation in KSHV-harboring cells. These finding suggests that ATLs can be expected to be a potential target for the treatment of KSHV or other similar virus infection related tumors and other diseases.

## Data Availability Statement

The original contributions presented in the study are included in the article/**Supplementary Material**. Further inquiries can be directed to the corresponding author.

## Author Contributions

W-YL and G-HZ conceived and designed the study, analyzed all the data and wrote the manuscript. W-YL performed all the experiments. YW helped with cell culture and some reporter assays. All authors contributed to the article and approved the submitted version.

## Conflict of Interest

The authors declare that the research was conducted in the absence of any commercial or financial relationships that could be construed as a potential conflict of interest.

## Publisher’s Note

All claims expressed in this article are solely those of the authors and do not necessarily represent those of their affiliated organizations, or those of the publisher, the editors and the reviewers. Any product that may be evaluated in this article, or claim that may be made by its manufacturer, is not guaranteed or endorsed by the publisher.

## Supplementary Material

Supplementary Figure 1 The overexpression of single ATL had no significant effect on the expression of other two ATL proteins. **(A)** The physiological expression of the ATL1/2/3 proteins in iSLK.rKSHV.219 cells and BCBL-1 cells. **(B–D)** iSLK.rKSHV.219 cells were transfected with different amounts of ATL1, ATL2, or ATL3 respectively. 48 hours later, protein levels of ATL1, ATL2, ATL3, and other ER-forming proteins were measured by western blot with indicated antibody.

Supplementary Figure 2 ATL proteins alter viral DNA replication in the cell. **(A, B)** iSLK.rKSHV.219 cells were transfected with vector, ATL1, ATL2 or ATL3 respectively. 48 hours later, mRNA levels of KSHV latency genes *vCyclin* and *vLANA* were measured by RT-qPCR, with normalization to *actin* using the ΔΔCT method. **(C, D)** iSLK.rKSHV.219 cells were transfected with siCtrl, siATL1/2/3#1, or siATL1/2/3#2 respectively. 48 hours later, mRNA levels of KSHV latency genes *vCyclin* and *vLANA* were measured by RT-qPCR, with normalization to *actin* using the ΔΔCT method. Data are presented as means of three technical replicates (n=3, group values are indicated by mean ± SEM; * p<0.05; ** p<0.01).

Supplementary Figure 3 The knockdown of single ATL had no significant effect on the expression of KSHV lytic genes. **(A, B)** iSLK.rKSHV.219 cells were transfected with siCtrl, siATL1, siATL2, or siATL3 respectively. 48 hours later, mRNA levels of KSHV lytic genes *ORF9* and *ORF50* were measured by RT-qPCR, with normalization to *actin* using the ΔΔCT method. **(C, D)** iSLK.rKSHV.219 cells were transfected with siCtrl, siATL1, siATL2, or siATL3 respectively followed by treatment with tetracycline plus valproate (VPA) for 48 hours to induce KSHV lytic reactivation. mRNA levels of KSHV lytic genes *ORF9* and *ORF50* were measured by RT-qPCR, with normalization to *actin* using the ΔΔCT method. **(E)** Knockdown efficiencies of siATL1/2/3 in iSLK.rKSHV.219 cells were measured by RT-qPCR. Data are presented as means of three technical replicates (n=3, group values are indicated by mean ± SEM; *p<0.05; **p<0.01).

Supplementary Figure 4 Rescue of single ATL in ATL1/2/3 knockdown cells could antagonize the inhibitory effect of ATL1/2/3 knockdown on the lytic reactivation of KSHV. iSLK.rKSHV.219 cells were transfected with siCtrl, siATL1/2/3^#1^ or siATL1/2/3^#1^ plus ATL1 **(A)**, ATL2 **(B)**, or ATL3 **(C)** respectively. 48 hours later, mRNA levels of KSHV lytic genes *ORF9* and *ORF50* were measured by RT-qPCR, with normalization to *actin* using the ΔΔCT method. **(D–F)** Knockdown and rescue efficiencies of ATL1/2/3 in iSLK.rKSHV.219 cells were also measured by RT-qPCR. Data are presented as means of three technical replicates (n=3, group values are indicated by mean ± SEM; *p<0.05; **p<0.01).

Supplementary Figure 5 The knockdown of single ATL had no significant effect on the ER stress. **(A–C)** iSLK.rKSHV.219 cells were transfected with siCtrl, siATL1, siATL2, or siATL3 respectively followed by treated with BFA for 24 hours. mRNA levels of ER stress related genes *BIP* and *CHOP* were measured by RT-qPCR, with normalization to *actin* using the ΔΔCT method. Data are presented as means of three technical replicates (n=3, group values are indicated by mean ± SEM; *p<0.05; **p<0.01). **(D–F)** The corresponding protein expression was detected by western blot with the indicated antibody.
