# Supplementary figures and images for: Endoplasmic Reticulum-Shaping Atlastin Proteins Facilitate KSHV Replication
Source: Front Cell Infect Microbiol. 2022 Jan 13;11:790243. doi: 10.3389/fcimb.2021.790243 (PMC8792907; doi:10.3389/fcimb.2021.790243)

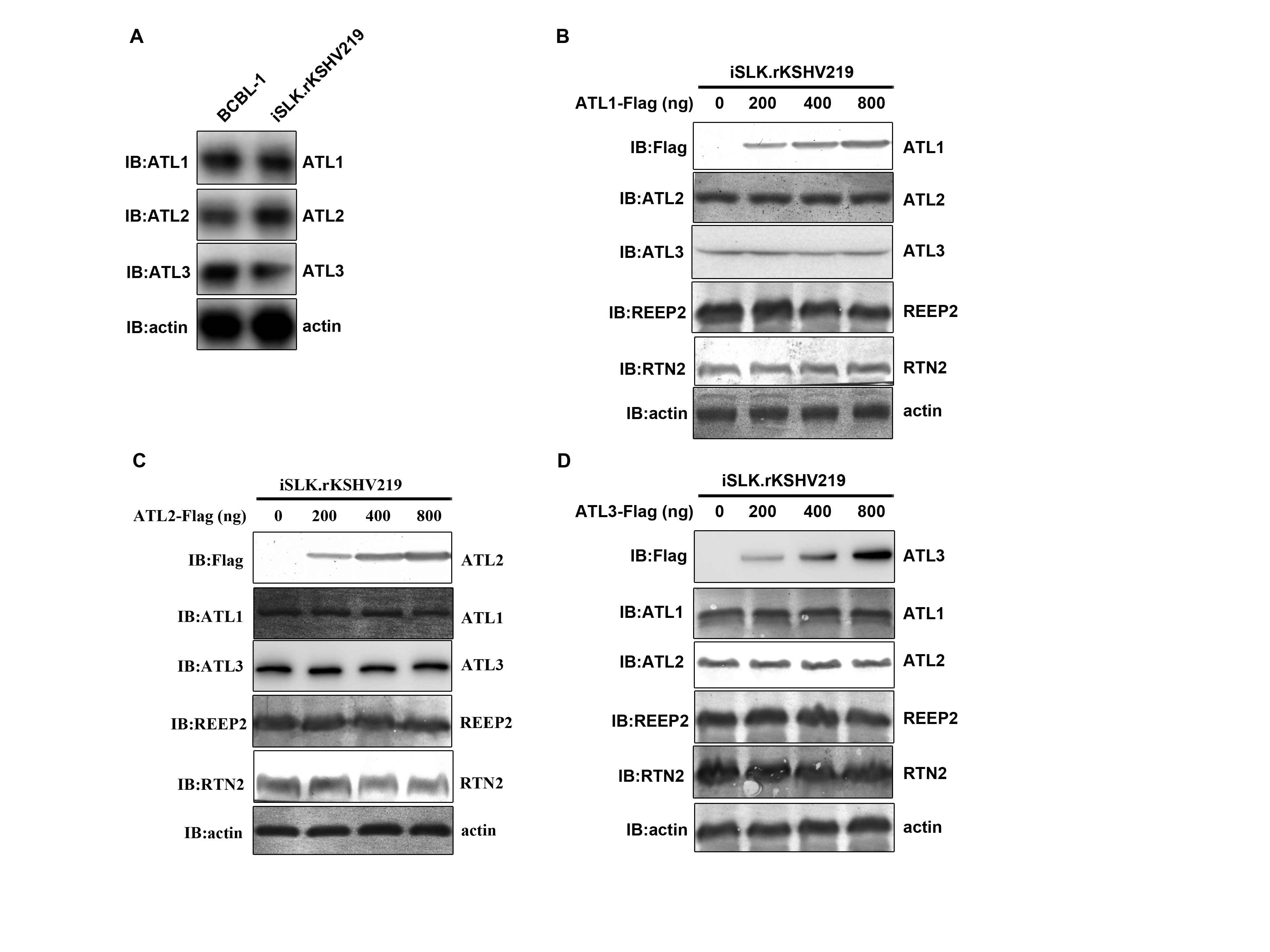

Supplement: Supplementary Figure 1 — The overexpression of single ATL had no significant effect on the expression of other two ATL proteins. (A) The physiological expression of the ATL1/2/3 proteins in iSLK.rKSHV.219 cells and BCBL-1 cells. (B–D) iSLK.rKSHV.219 cells were transfected with different amounts of ATL1, ATL2, or ATL3 respectively. 48 hours later, protein levels of ATL1, ATL2, ATL3, and other ER-forming proteins were measured by western blot with indicated antibody. [file Image_1.jpeg]

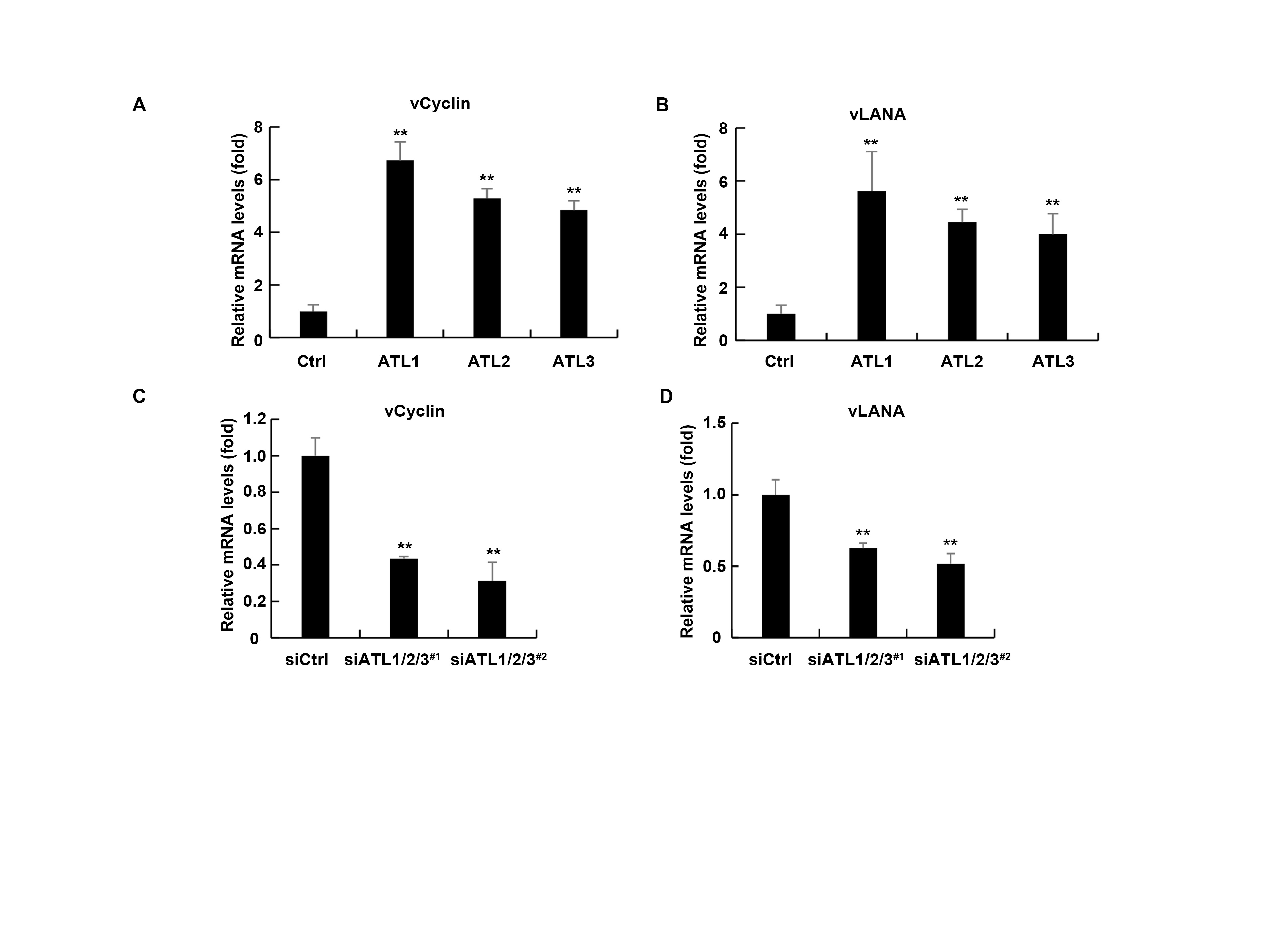

Supplement: Supplementary Figure 2 — ATL proteins alter viral DNA replication in the cell. (A, B) iSLK.rKSHV.219 cells were transfected with vector, ATL1, ATL2 or ATL3 respectively. 48 hours later, mRNA levels of KSHV latency genes vCyclin and vLANA were measured by RT-qPCR, with normalization to actin using the ΔΔCT method. (C, D) iSLK.rKSHV.219 cells were transfected with siCtrl, siATL1/2/3#1, or siATL1/2/3#2 respectively. 48 hours later, mRNA levels of KSHV latency genes vCyclin and vLANA were measured by RT-qPCR, with normalization to actin using the ΔΔCT method. Data are presented as means of three technical replicates (n=3, group values are indicated by mean ± SEM; * p<0.05; ** p<0.01). [file Image_2.jpeg]

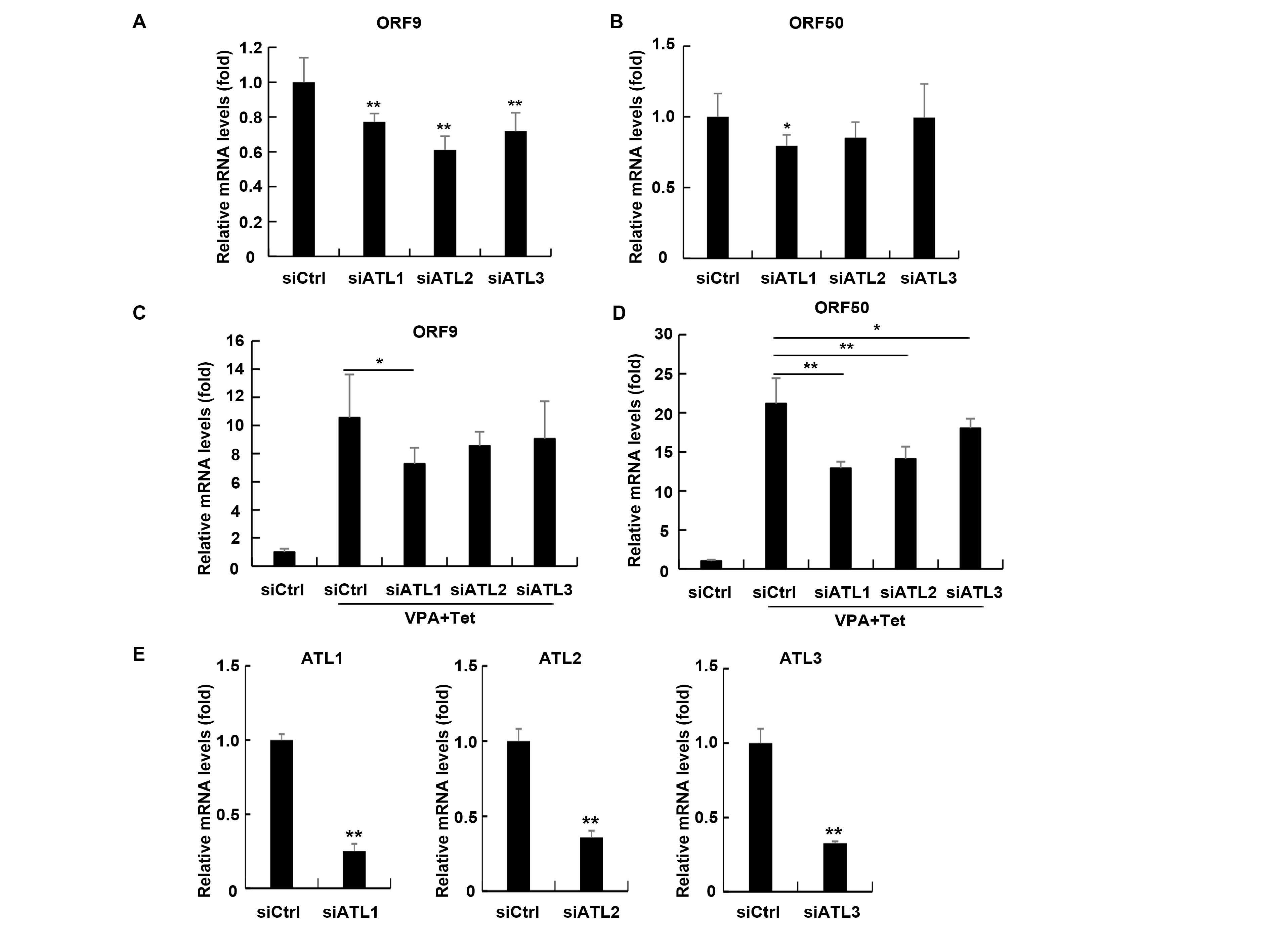

Supplement: Supplementary Figure 3 — The knockdown of single ATL had no significant effect on the expression of KSHV lytic genes. (A, B) iSLK.rKSHV.219 cells were transfected with siCtrl, siATL1, siATL2, or siATL3 respectively. 48 hours later, mRNA levels of KSHV lytic genes ORF9 and ORF50 were measured by RT-qPCR, with normalization to actin using the ΔΔCT method. (C, D) iSLK.rKSHV.219 cells were transfected with siCtrl, siATL1, siATL2, or siATL3 respectively followed by treatment with tetracycline plus valproate (VPA) for 48 hours to induce KSHV lytic reactivation. mRNA levels of KSHV lytic genes ORF9 and ORF50 were measured by RT-qPCR, with normalization to actin using the ΔΔCT method. (E) Knockdown efficiencies of siATL1/2/3 in iSLK.rKSHV.219 cells were measured by RT-qPCR. Data are presented as means of three technical replicates (n=3, group values are indicated by mean ± SEM; *p<0.05; **p<0.01). [file Image_3.jpeg]

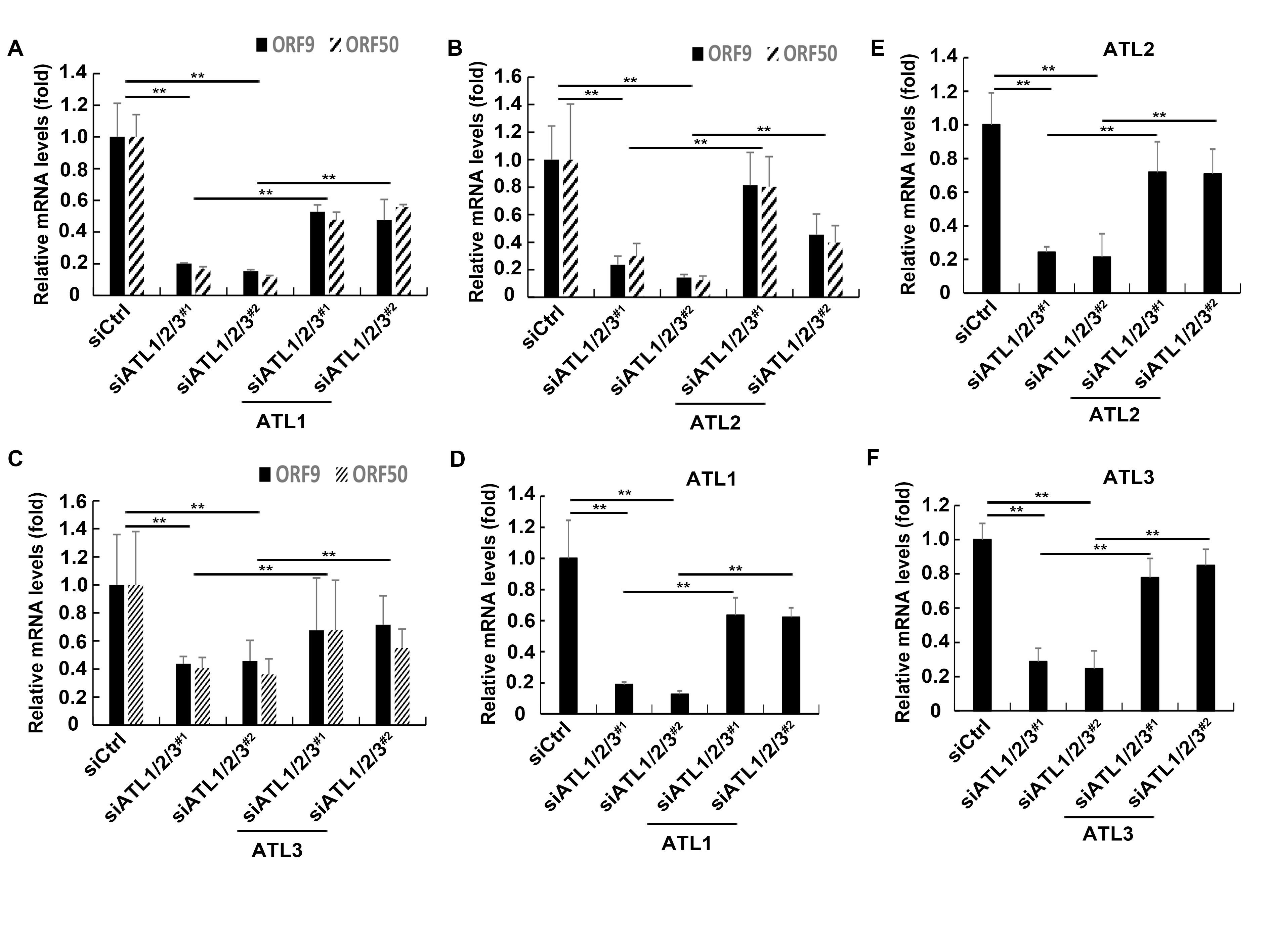

Supplement: Supplementary Figure 4 — Rescue of single ATL in ATL1/2/3 knockdown cells could antagonize the inhibitory effect of ATL1/2/3 knockdown on the lytic reactivation of KSHV. iSLK.rKSHV.219 cells were transfected with siCtrl, siATL1/2/3#1 or siATL1/2/3#1 plus ATL1 (A), ATL2 (B), or ATL3 (C) respectively. 48 hours later, mRNA levels of KSHV lytic genes ORF9 and ORF50 were measured by RT-qPCR, with normalization to actin using the ΔΔCT method. (D–F) Knockdown and rescue efficiencies of ATL1/2/3 in iSLK.rKSHV.219 cells were also measured by RT-qPCR. Data are presented as means of three technical replicates (n=3, group values are indicated by mean ± SEM; *p<0.05; **p<0.01). [file Image_4.jpeg]

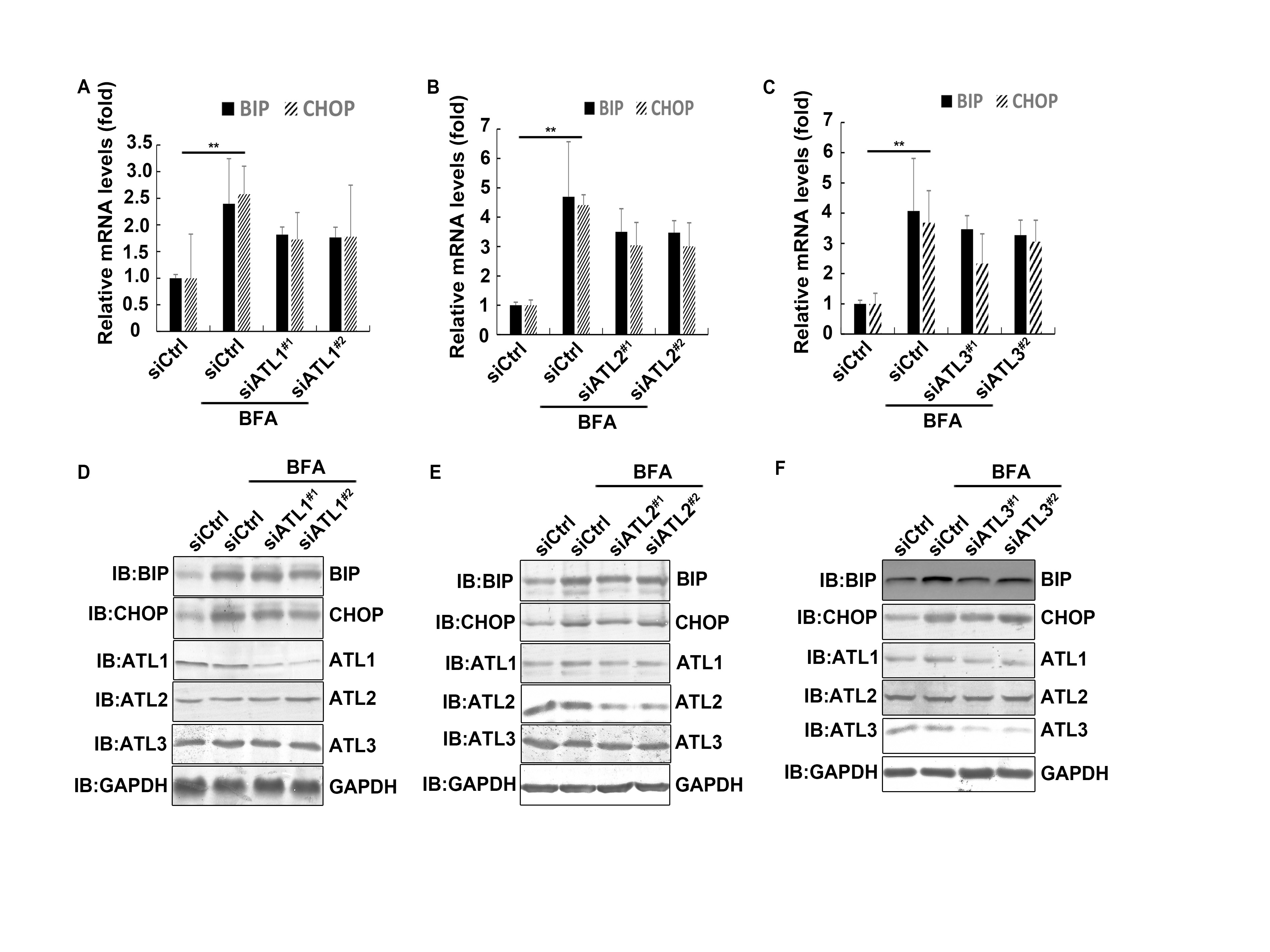

Supplement: Supplementary Figure 5 — The knockdown of single ATL had no significant effect on the ER stress. (A–C) iSLK.rKSHV.219 cells were transfected with siCtrl, siATL1, siATL2, or siATL3 respectively followed by treated with BFA for 24 hours. mRNA levels of ER stress related genes BIP and CHOP were measured by RT-qPCR, with normalization to actin using the ΔΔCT method. Data are presented as means of three technical replicates (n=3, group values are indicated by mean ± SEM; *p<0.05; **p<0.01). (D–F) The corresponding protein expression was detected by western blot with the indicated antibody. [file Image_5.jpeg]
